# Key aspects of nursing practice that underpin certain discourses: an integrative review

**DOI:** 10.1590/0034-7167-2025-0050

**Published:** 2026-06-12

**Authors:** María Liliana Calderón Macías, Samantha Dayanara Álvarez Basurto, Jennifer Alexandra Rivas Zambrano, Jessica Tatiana González Quiroz

**Affiliations:** I Universidad Estatal del Sur de Manabí. Jipijapa, Manabí, Ecuador; II Pontificia Universidad Católica del Ecuador. Quito, Pichincha, Ecuador

**Keywords:** Nursing, Professional Autonomy, Gender Role, Education, Nursing, Curriculum., Enfermagem, Autonomia Profissional, Papel de Gênero, Educação Continuada em Enfermagem, Currículo.

## Abstract

**Objectives:**

to synthesize the key aspects of nursing upon which some discourses that shape its development are based.

**Methods:**

a two-stage search strategy was designed. First, searches were conducted in specific databases focusing on nursing literature. Subsequently, studies addressing the objective of this review were identified and screened. The techniques used included following citations, searching for subsequent publications, and using reference lists to conduct a reverse search.

**Results:**

thirty-one texts were analyzed and categorized into four dimensions: Professional autonomy; Patriarchal gender issues; The Nightingale School of Nursing and Midwifery; and Curriculum reforms.

**Conclusions:**

the aspects summarized in this review, through the principles they promote or their underlying messages, have either contributed to the professional and disciplinary development of nursing, or conversely, have hindered nursing from being recognized by society as a professional discipline.

## RESUMO

**Objetivos:**

sintetizar os principais aspectos da enfermagem que fundamentam alguns dos discursos que determinam seu desenvolvimento.

**Métodos:**

foi elaborada uma estratégia de busca em duas etapas. Primeiramente, foram realizadas buscas em bases de dados específicas, com foco na literatura de enfermagem. Posteriormente, foram identificados e rastreados estudos que abordassem o objetivo desta revisão. As técnicas utilizadas incluíram rastreamento de citações, busca por publicações subsequentes e uso de listas de referências para retrocesso.

**Resultados:**

31 textos foram analisados e categorizados em quatro dimensões: Autonomia profissional; Questões patriarcais de gênero; A escola de Nightingale; e Reformas curriculares.

**Conclusões:**

os aspectos resumidos nesta revisão, pelos princípios que promulgam ou pelos seus discursos ocultos, têm buscado o desenvolvimento profissional e disciplinar da enfermagem ou, pelo contrário, não permitem que a enfermagem seja socialmente reconhecida como disciplina profissional.

## INTRODUCTION

Michel Foucault’s first lecture as a professor at the *Collège de France* in 1970 was entitled “The Order of Discourse”^(1)^. During this keynote lecture, the scholar stated: “... in every society, the production of discourse is simultaneously controlled, selected, and redistributed by a certain number of procedures whose function is to manage its power and potential dangers, to control unpredictable events, and to circumvent its heavy and formidable materiality”^(2)^. Following the same line of reasoning, Foucault^(2)^ mentions that within any discourse, there is “will to truth” hidden, which he explains as the various social structures or practices that create and sustain the production of knowledge, through which meanings are transmitted and systems of thought and knowledge are reflected. Based on the aforementioned Foucault’s postulates, the literature suggests that discursive formations allow us to define conceptual possibilities and, in advance, determine both what can and cannot be said, and subsequently, what can be thought within a specific context or period of time^(3)^.

Furthermore, Foucault^(2)^ argues that true discourse underwent a “historical shift”. In the 6^th^ century, true discourse-“the dominant discourse, uttered by those who had the right to speak”-was the discourse that administered justice and not only anticipated changes but also contributed to their realization^(2)^. Foucault^(2)^ argues that, a century later, truth no longer resided in the discourse itself, nor in a person who uttered it, but rather in what the discourse said-“its meaning, its form, its subject matter”-or what it referred to. In this regard, discourse could no longer be seen as inherently linked to the exercise of power. Following Foucault^(2)^, these historical circumstances-within the realm of discursive production-are constantly shifting, leading to discoveries that come to be established and recognized as “new forms of truth”. For a discourse to be established as a “will to truth”, Foucault^(2)^ states that it must be supported by an “institutional foundation”. This foundation-in the author’s words-“is reinforced and accompanied by a series of practices such as pedagogy, the book publishing system, editing, libraries, learned societies of the past, and modern laboratories”^(2)^. However, it is also essential that this initiative addresses a social need to ensure its relevance, dissemination, and equitable distribution among members of society.

Researchers in this field illustrate this point from an academic perspective. These authors argue that what can be said about nursing knowledge is classified according to the underlying concepts and regulatory systems that govern and determine it, thus demonstrating a sufficient level of understanding within the bounds of acceptable scholarly discourse^(3)^. Additionally, they point out that, like other disciplines, nursing also has established rules for conceptualizing its knowledge requirements, and that, historically, research conducted from a Foucault’s perspective has made it possible to visualize the internal structure of what is known as nursing knowledge^(3)^. According to these authors, it is through this type of research that it has been possible to uncover the dominant discourses that have shaped nursing education and determined the skills and competencies required for its practical application^(3)^.

Following Foucault’s approach, the interest in studying discourses-given their role in shaping human consciousness and their ability to reveal the systems of thought and knowledge that influence social practices^(4)^-focuses on the practices that, in this case, have shaped the development of nursing as a professional discipline. From this perspective, and in accordance with Springer & Clinton^(3)^, research of this nature critically examines the complexities of discourse, its emergence and resistance, its content, and the contexts that determine it, and attempts to understand not only what is known about a phenomenon, but also what remains unexpressed, whether due to ignorance or deliberate concealment. In this context, this review aims to answer the question: what are the key aspects of nursing that underpin the discourses shaping its development as a professional discipline?

## OBJECTIVES

To synthesize the key aspects of nursing that form the basis of some discourses that determine its development.

## METHODS

This is an integrative literature review that aimed to identify, analyze, and synthesize findings from scientific evidence, in order to generate new knowledge on the topic. The results are part of a doctoral dissertation, the protocol of which was approved by the Research Ethics Committee of the School of Nursing at the *Universidad de Antioquia*.

**Figure 1:**
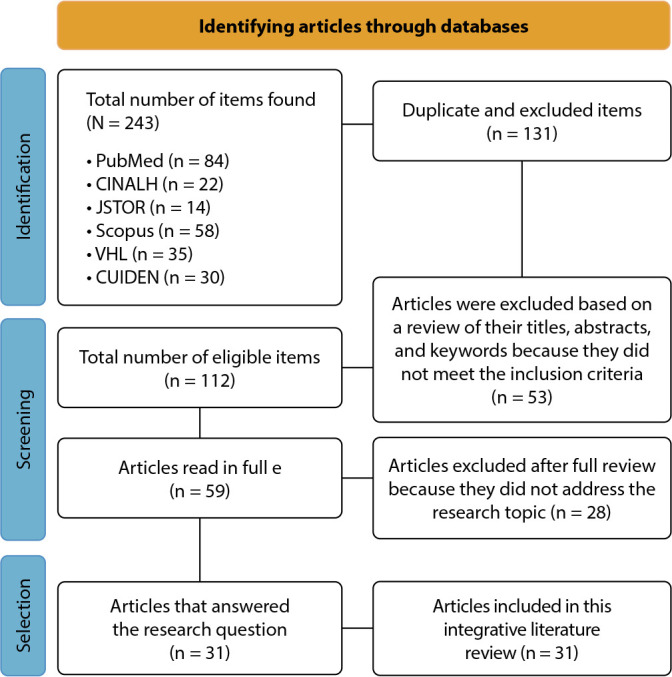
Flowchart of the study selection process according to Preferred Reporting Items for Systematic Reviews and Meta-Analyses, 2022

To conduct this review, a search strategy was designed that followed the recommendations of the Spanish adaptation of the Preferred Reporting Items for Systematic Reviews and Meta-Analyses statement. The databases included were PubMed, CINAHL, JSTOR, and Scopus, for English-language articles, and the Virtual Health Library and CUIDEN, for Spanish-language articles. Mendeley was used as the reference management software. The search terms included the keywords “nursing”, “nursing education”, “professional autonomy”, and “gender role”, using the Boolean operator “AND”. The second stage involved identifying and locating key texts that addressed the objectives of this review. The techniques used in this stage included screening citations, searching for subsequent publications, and using reference lists to work backwards. The titles were initially examined to determine their relevance. Exclusions were made based on study objectives and inclusion and exclusion criteria. Literature in Spanish, Portuguese, and English was included, regardless of publication year. Articles whose full text was not freely available for online access and download, and any duplicate entries, were excluded. A total of 31 articles were identified, and the selection process is shown in Figure 1.

## RESULTS

Available information on the central topic of this review dates back to the last 20 years, with 2021 being the year with the highest number of publications. Most literature was published by authors from Brazil and Colombia, with Brazilian journals being the most prolific in this area. Concerning study design, theoretical reflections and qualitative studies predominated, and two cross-sectional studies were also included. Four book chapters and one doctoral thesis, all of which offer a historical-critical perspective on the topic, were also included. Chart 1 synthesizes this information.

**Chart 1:** Description of studies included in systematic review, 2024

N^o^.	Authors	Article title	Text type	Category	Year
1	Leddy S, Pepper JM^(5)^	*Socialización para el ejercicio profesional*	Book	I	1989
2	Romero MN^(6)^	*El Modelo Pedagógico en Enfermeria: Una proyección del papel social de la mujer*	Theoretical reflection	I	1992
3	Aponte Garzón LH^(7)^	*Aspectos pedagógicos en la formación del recurso de enfermería en Colombia*	Theoretical reflection	I	1997
4	Cano-Caballero Gálvez MD^(8)^	*Enfermería y género Tiempo de reflexión para el cambio*	Theoretical reflection	I	2004
5	Varjus SL, Leino-Kilpi H, Suominen T^(9)^	Professional autonomy of nurses in hospital settings - a review of the literature	Integrative review	I	2011
6	Watkins C, Hart PL, Mareno N^(10)^	The effect of preceptor role effectiveness on newly licensed registered nurses perceived psychological empowerment and professional autonomy	Cross-sectional correlational study	I	2016
7	Velandia Mora AL^(11)^	*Historia de la enfermería en Colombia*	Book	I	2016
8	Mena Tudela D, González Chordá VM^(12)^	*Imagen social de la enfermería, ¿estamos dónde queremos?*	Theoretical reflection	I	2018
9	Oshodi TO, Bruneau B, Crockett R, Kinchington F, Nayar S, West E^(13)^	Registered nurses’ perceptions and experiences of autonomy: A descriptive phenomenological study	Phenomenological analysis	I	2019
10	Franco Coffré JA^(14)^	*Percepción social de la profesión de enfermería*	Theoretical reflection	I	2019
11	Pursio K, Kankkunen P, Sanner-Stiehr E, Kvist T^(15)^	Professional autonomy in nursing: An integrative review	Integrative review	I	2021
12	Webler N, Almeida LCG de, Carneiro JB, Campos LM, Glaeser TA, Couto TM^(16)^	Professional autonomy in dealing with complications: discourse of obstetric nurses working in planned home births	Qualitative study	I	2023
13	Matejski MP^(17)^	Nursing education, professionalism, and autonomy: social constraints and the Goldmark Report	Theoretical reflection	II	1981
14	Marques Lopes MJ, Cezar Leal SM^(18)^	*A feminização persistente na qualificação profissional*	Theoretical reflection	II	2005
15	Ayala-Valenzuela R^(19)^	*Biopoder: el poder y la violencia en la formación de profesionales de enfermería*	Theoretical reflection	II	2008
16	Lemos De Souza L, Borges Araújo D, Souza Silva D, Cristina V, Bêrredo M^(20)^	*Representações de gênero na prática de enfermagem na perspectiva de estudantes*	Cross-sectional study	II	2014
17	Nogueira I, Spagnol G, Rocha F, Lopes MH, Marques D, Santos D^(21)^	Gender and Empowerment by Nursing Students: Representations, Discourses and Perspectives	Discourse analysis	II	2023
18	Nigthingale F^(22)^	Notes on nursing: What it is, and what it is not	Book	III	1860
19	Cohen B^(23)^	Florence Nigthingale	Theoretical reflection	III	1984
20	Castrillón Agudelo MC^(24)^	*La Dimensión Social de la Práctica de la Enfermería: Historia y sociología de la enfermería*	Book	III	2012
21	Riegel F, Crossetti M da GO, Martini JG, Nes AAG^(25)^	Florence Nightingale’s theory and her contributions to holistic critical thinking in nursing	Theoretical reflection	III	2021
22	Diogo PMJ, Freitas BHBM de, Costa AIL da, Gaíva MAM^(26)^	Care in pediatric nursing from the perspective of emotions: from Nightingale to the present	Theoretical reflection	III	2021
23	Breigeiron MK, Vaccari A, Ribeiro SP^(27)^	Florence Nightingale: Legacy, present and perspectives in COVID-19 pandemic times	Theoretical reflection	III	2021
24	McCauley L, Hayes R^(28)^	From Florence to fossil fuels: Nursing has always been about environmental health	Theoretical reflection	III	2021
25	Manfredy M^(29)^	*Bases teóricas para la estructuración curricular*	Book	IV	1980
26	Pan American Health Organization^(30)^	*Análisis prospectivo de la educación en enfermería*	Book	IV	1989
27	Posada Vera EM^(31)^	*Discursos sobre la configuración de la enfermería como disciplina o como profesión: un estudio de caso en una facultad de enfermería*	Doctoral thesis	IV	2015
28	Petry S, Padilha MI, Costa R, Mancia JR^(32)^	Curricular reforms in the transformation of nursing teaching in a federal university	Single case study	IV	2020
29	Buchanan C, Howitt ML, Wilson R, Booth RG, Risling T, Bamford M^(33)^	Predicted Influences of Artificial Intelligence on Nursing Education: Scoping Review	Scoping review	IV	2021
30	Nogueira IC, Santos D de S, Sanfelice CF de O, Silva EM, Assis AESQ^(34)^	Gender debate as a challenge in nursing training	Qualitative study	IV	2021
31	Duque PA, Flórez-Pulido LM, Mejía-Ramírez LF^(35)^	*Revisión de literatura integradora del conocimiento disciplinar de la enfermería y el currículo*	Theoretical reflection	IV	2024

From the 31 selected texts, four categories emerged that encompass key aspects of nursing upon which some discourses about this profession are based. These are: Professional autonomy (12 texts); Patriarchal gender issues (5 texts); The Nightingale School of Nursing and Midwifery (7 texts); and Curriculum reforms (7 texts).

## DISCUSSION

### Category I - Professional autonomy

The first key aspect of nursing is the constant pursuit of professional autonomy, an element that Leddy & Pepper^(5)^ consider fundamental for achieving professional status. For Pursio *et al*.^(15)^, professional autonomy is related to nursing staff participation in decision-making and their ability to influence work practices. According to this author, regarding autonomy in nursing, two broad dimensions are recognized: autonomy in clinical practice and autonomy in professional practice. Oshodi *et al*.^(13)^ define clinical autonomy as “the ability to act beyond standard practice and make individual decisions regarding patient care”.

According to Varjus *et al*.^(9)^ and Watkins *et al*.^(10)^, professional autonomy, in addition to the aforementioned aspects, encompasses, respectively, nurses’ ability to “develop processes to improve the quality of nursing care and patient safety” and “their ability to influence work practices and conditions”. Pursio *et al*.^(15)^, in their integrative review on autonomy in nursing, identified the following as essential factors for the development of professional autonomy among nurses: “shared leadership, professional competencies, interprofessional and intraprofessional collaboration, and a healthy work environment”. However, they also noted that the historically subordinate role of nursing to the medical profession exacerbates the gap in interprofessional collaboration and reinforces unequal roles within the healthcare workforce.

Along the same lines of discussion, Latin American authors such as Aponte^(7)^, Romero^(6)^, and Velandia^(11)^ agree that, behind the discourse of vocation and religious morality-which is often associated with the role of being a woman-lie power dynamics with those in positions of higher authority, which place nursing in a permanent state of subordination. This situation directly hinders the social, ideological, educational, and professional advancement that nursing has sought to achieve for nearly two centuries. Cano^(8)^ identifies as a cause of the lack of autonomy in the nursing profession that its members still lack a clear definition of what constitutes “care”, and consequently, they have not fully understood or internalized the unique and independent functions that this profession has as a discipline. Mena & González^(12)^, Franco^(14)^, and Webler *et al*.^(16)^ agree with this, stating that, in the healthcare field, society currently perceives male physicians as representing strength, authority, and knowledge, while nurses continue to be stereotyped as the female figure-weak, submissive, and marginalized-who depends on the physician to practice their profession. Building on Aponte’s^(7)^ and Romero’s^(6)^ work, this socially constructed structure has not only regulated the profession practice, but has also influenced training processes, shaping a distinct identity for nursing: one that is predominantly female, characterized by a religious and vocational calling, disciplined and rule-oriented behavior, and a tendency to subordinate itself to the biomedical model.

### Category II - Patriarchal gender issues

Several authors consider that patriarchal gender issues are a fundamental and central problem that continues to influence the profession^(16)^. Historically, nursing has been associated with care, and care, in turn, has always been conceived as a feminine practice. De Souza *et al*.^(20)^ point to Nightingale as being responsible for establishing modern nursing based on moral and religious principles that have persisted until the present day and have promoted the gender division of labor since the mid-19^th^ century. According to these authors, this gender division is closely linked to the social division of labor between medicine and nursing, as this differentiation is similar to that between science and art, study and vocation, and intellectual and manual labor. These differences are not limited to patriarchal gender issues, but also encompass issues of race, class, religion, age, and other aspects of social structure^(20)^.

Foucault^(36)^, a philosopher who studied discourse and the power that operates behind it, argues that power is exercised through relationships, and that it not only has a dominant character, but also manifests itself as a productive power directed at the body, aiming to train and shape it according to its own purposes. Therefore, issues related to religion, gender, classism, and patriarchal culture, which have historically permeated nursing, play a significant role in shaping nursing knowledge and practice. This is demonstrated by De Souza^(20)^ in his observational study on gender discourse among nursing students, in which he identified the use of everyday language in their social interactions that perpetuates gender stereotypes, portraying them as authoritarian, kind, submissive, angelic, devout medical assistants, and using other more derogatory terms, such as “prostitute” or “homosexual”. According to Marques & Cezar^(18)^, the perpetual dichotomy between medicine practice (treatment) and nursing practice (care) clearly demonstrates the power of men over women, as evidenced by the lack of social recognition for nursing, the imposition of longer working hours, and the payment of lower wages. Similarly, Nogueira *et al*.^(21)^ argue that these practices, in addition to reinforcing gender stereotypes and marginalizing nursing professionals, contribute to the perpetuation of the biomedical model within the dominant neoliberal system, which is more concerned with treating diseases than with refocusing its efforts towards developing a healthier society.

As has been seen, the development of nursing as a profession and discipline has been subject to power dynamics that have permeated this process for over 200 years. According to Ayala^(19)^, in nature, power manifests itself in various forms and is propagated through classic human relationships such as: man-woman, father-child, physician-patient, professor-student, etc. Following Foucault’s approach^(37)^, this author defines power as “the several forms of domination that can be exercised within society”, and identifies academic or educational spaces, hospitals, churches, and prisons^(36)^ as unparalleled settings for the exercise and reproduction of power relations. Although these spheres have entirely different dynamics, the power relations that exist within them are consolidated, legitimized, and perpetuated through a similar mechanism: discursive practices^(19)^. Regarding the above, Ayala^(19)^ mentions that the differentiation between the dynamics of power structures is determined by two factors: first by the interpersonal relationships among the members of that group (school, hospital, prison, etc.); and second by the closed-system nature of the institution within which these structures are created. Similarly, Deleuze^(38)^ - another disciple of Foucault - argues that human beings experience power relations throughout their lives: first within the family, then within school, if male, within the military, occasionally in hospitals, and in some cases, in prison^(38)^.

According to Foucault^(36)^, in power relations, emphasis is placed on the intentional domination and manipulation of the human body, “which is shaped, trained, made to obey, made to respond, becomes skilled, or whose strength is multiplied”. Ayala^(19)^, in his reflections on biopower, argues that, since classical times, the body has been the subject of study with the aim of “knowing it, understanding it, and making it intelligible”. From a biopolitical perspective, Foucault^(36)^ examines the body within a framework of subjugation, exercised through military, school, or hospital regulations established to control or intervene in its operational practices. According to Ayala^(19)^, education plays a fundamental role in shaping bodies, applying its disciplinary norms within a context of constant coercion. In Foucault’s words^(36)^, “disciplinary control does not consist simply in teaching or imposing a series of specific gestures; it imposes the optimal relationship between a particular gesture and the overall posture of the body, which is the condition for its effectiveness and efficiency”. To illustrate the historical prevalence of these types of practices in nursing education, Matejski^(17)^, in her research on education, professionalism, and autonomy in nursing, highlights a passage from the account of the first 40 years of the Johns Hopkins Hospital School of Nursing: “The nurse is a soldier. Absolute and unconditional obedience is the fundamental principle of the military system... rigor and precision produce better nurses”.

### Category III - The Nightingale School of Nursing and Midwifery

With the creation of the first nursing training program, led by Florence Nightingale, a training model was established which, according to several authors, is still evident in some curricula in the region and continues to play a significant role in nursing consolidation as a professional discipline. Regarding this, Cohen^(23)^ states: “The aim of this school was to train nurses capable of training others. The nurses who would graduate from this school would not be primarily involved in patient care, but would instead be tasked with managing hospitals and public institutions, in order to raise nursing care status”.

According to this author, the Nightingale School of Nursing and Midwifery, established in 1859, was based on three principles: first, aspiring nurses received one year of theoretical and practical training in hospitals; second, these students were required to live in a residence where their conduct and discipline could be closely supervised; and third, this residence had to be located near the hospital so that students would be available to provide services at any time, thus allowing them to learn by doing^(23)^. For Castrillón^(24)^, despite seeming like radical principles for that era, in many schools these principles were maintained and perpetuated for over a century, or at least until the establishment of a professional training system for nursing. However, Riegel *et al*.^(25)^ argue that Nightingale’s legacy is crucial for addressing knowledge gaps related to the holistic dimension of nursing care, which ultimately leads to more effective clinical decision-making by students. Breigeiron *et al*.^(27)^ and Diogo *et al*.^(26)^ agree with this and conclude that this model was fundamental to nursing consolidation.

Another important contribution to the development of nursing practice was the famous nursing notes written by Florence Nightingale. In these notes, she defined nursing as “the art of maintaining health, preventing illness, or recovering from illness”^(22)^. Regarding this, there is an established theory, the environmental or ecological theory, which, according to McCauley & Hayes^(28)^, describes ten factors that influence health and that should be taken into consideration in nursing care. Additionally, these notes also recognize the need to establish training centers to educate specialized staff, both for the care of hospitalized patients and for those requiring other types of specialized care, such as older adults and people with disabilities^(22)^. For Castrillón^(24)^, this particular model of thinking and training has left a lasting legacy in the nursing profession, resulting in a larger number of nurses working in hospital settings-characteristic of a healthcare system with a greater emphasis on curative care-and representing the ideal of many nursing students.

### Category IV - Curriculum reforms

According to Castrillón^(24)^, no other university program has undergone as many curriculum changes-driven by public and educational policy discourse-as the nursing program. According to this author, nursing is the most susceptible practice to changes in its functions and activities in response to transformations related to the division of labor within the healthcare sector and the continuous influx of technical or non-professional staff^(24)^. Castrillón^(24)^ believes that current nursing training models are more focused on producing public service workers than on preparing professional nurses.

Manfredy^(29)^ identified four stages that characterized nursing education in Latin America between 1940 and 1980. Manfredy notes that in the 1940s, the curricula developed for this training were structured around isolated, independent courses, lacked any connection to the healthcare sector or higher education institutions, and relied on the traditional lecture method, whose aim was to mold students into replicas of their professors^(29)^.

As early as the 1950s, in this region, training programs were characterized by a curriculum structure that divided subjects into basic and clinical courses; the academic levels were organized on an annual basis; and the teaching methodology-very similar to that of the past-was combined with hands-on, demonstrative teaching using simulators or in specially equipped laboratories^(29)^. Since the early 1960s, following a new change in the curriculum structure, the distribution of subjects reflected a more holistic approach to learning, incorporating the teaching of social sciences and humanities. Additionally, there was growing interest in equipping school libraries with materials and practical guides for clinical and community practice, tailored to the region’s specific needs^(29)^. Finally, between 1970 and 1980, curriculum changes addressed two main issues: first, the need to develop a professional profile that met each country’s specific needs in the region, not only in terms of general health, but also in terms of family and community health, i.e., primary healthcare; and second, the development of profiles that would facilitate the integration of teaching and clinical practice, thus promoting the development of both clinical and community-based practices^(29)^.

Castrillón^(24)^ takes the analysis of curricular transformations in nursing a step further, examining developments up to the end of the 20^th^ century. According to this author, starting in the 1980s, and thanks to the movement initiated by the Alma-Ata Declaration of 1978, several strategies were implemented to strengthen professional training programs for nurses^(24)^. Among other key objectives are: reorienting the teaching of social sciences to reflect the country’s socio-health realities; innovating clinical practices; strengthening community health promotion activities; and enhancing the development of skills related to physical assessment. Concerning the planning and implementation of healthcare services to ensure comprehensive healthcare, in short, as Castrillón^(24)^ stated, “the aim was to develop a comprehensive training program whose curriculum would be based on the specific needs of the country”. By the late 1990s-as part of the development of specific policies for higher education-the same author notes that universities began a process of transformation, seeking to contribute to society not only through teaching, but also through the generation of new knowledge as a result of scientific research^(24)^.

In nursing, in particular, these recent transformations led to the development of research areas, which subsequently supported: the creation of research groups to conduct and present this research; the need to develop specific graduate courses for nursing practice; participation in multidisciplinary projects; and establishment of framework agreements not only to expand clinical practice settings, but also to facilitate the exchange of academic experiences between both faculty and students^(24)^. In Castrillón’s words, “all of this led to a reconfiguration of the teaching and healthcare processes”.

The last decade of the 20^th^ century was characterized by the implementation of intervention plans, not only for curricula but also for the infrastructure of nursing schools, as a result of the retrospective analysis carried out - with the support of the Pan American Health Organization (PAHO) - on several nursing schools in ten Latin American countries^(30)^. The proposed interventions included developing educational and research projects to address local health issues and challenges within nursing; the need to establish international academic networks where key stakeholders in the profession could engage in scholarly discussions about the development of nursing knowledge; and the related aspects surrounding this knowledge^(30)^. Additionally, there was a call to increase the number of scientific publications, to promote participation in academic events, and to develop continuing education programs for graduates. According to Posada^(31)^, all of the aforementioned elements are key to consolidating nursing as a professional discipline. Hence, following Castrillón^(24)^, in Latin America, this process began to become evident for nursing starting in the 1990s.

Castrillón^(24)^ concludes by stating that the PAHO has played a central role in proposing and promulgating the policies and guidelines upon which most of these curriculum reforms have been based. The author also argues that these curriculum changes have not occurred simultaneously or from the same perspectives across all regions and countries, and that this is a result of the numerous debates, interpretations, and even resistance that the topic has generated^(24)^. Regarding this, Petry *et al*.^(32)^ state that the curriculum structure and the ongoing reforms in nursing education are shaped by the historical, political, epidemiological, and social context that the profession needs to meet society’s and job market’s demands.

An example of this is the post-pandemic context in which society currently finds itself. According to Buchanan *et al*.^(33)^, there is an urgent need for curriculum reform in nursing education programs, which should include, in their graduate profiles, the skills and competencies necessary to practice safe and efficient patient care in the digital age and with the use of artificial intelligence in healthcare systems. Additionally, Duque *et al*.^(35)^ argue that it is essential for curricula to incorporate the discipline’s metaparadigm in order to guide teaching and learning processes towards developing a nursing profile that gains greater recognition, and that supports nursing practice based on the scientific method and the specific knowledge frameworks of nursing. Finally, and in line with the categories mentioned above, Nogueira *et al*.^(34)^ assert that transforming nursing education processes is urgent, and that, currently, the analysis of gender issues and their direct influence on professional practice should also be considered as a key transversal element of the curriculum.

### Study limitations

The main limitation of this review lies in the geographical coverage of selected studies. Further research is needed to explore the topic of nursing development and its key aspects, particularly from the perspectives of the Anglo-Saxon and Asian regions, where significant nursing research is currently being conducted.

### Contributions to nursing, health, or public policy

According to Foucault, discourses mask a power dynamic that, while enabling certain things, also restricts others. In this sense, synthesizing the key aspects of nursing upon which certain discourses in this profession are based, becomes a framework for identifying the elements that need to be addressed to promote greater professional and disciplinary development in nursing, as well as identifying aspects of training that could be modified for the same purpose.

## CONCLUSIONS

The literature review reveals some aspects that, since the origins of modern nursing, have formed the basis for some of the discourses that have been implemented and circulated throughout the history of nursing, and which have been key factors in nursing education and nursing knowledge dissemination. Among other topics, the literature highlights the pursuit of professional autonomy, patriarchal gender issues, the Florence Nightingale School of Nursing and Midwifery, and curriculum reforms which, through the principles they espouse or their underlying motives, have either sought to promote the professional and disciplinary development of nursing, or conversely, have hindered nursing from achieving professional status and social recognition.

## Data Availability

The research data are available in a repository: https://preprints.scielo.org/index.php/scielo/preprint/view/11144/version/11750.
