# Supplementary material for: Key aspects of nursing practice that underpin certain discourses: an integrative review
Source: Rev Bras Enferm. 2026 Jun 12;79:e20250050. doi: 10.1590/0034-7167-2025-0050 (PMC13262981; doi:10.1590/0034-7167-2025-0050)
Supplement: Translation

# Translation: Aspectos claves de la enfermería sobre los que se soportan algunos discursos: una revisión integrativa

**DOI:** 10.1590/0034-7167-2025-0050es

**Authors:** María Liliana Calderón Macías, Samantha Dayanara Álvarez Basurto, Jennifer Alexandra Rivas Zambrano, Jessica Tatiana González Quiroz

**Affiliations:** I Universidad Estatal del Sur de Manabí. Jipijapa, Manabí, Ecuador; II Pontificia Universidad Católica del Ecuador. Quito, Pichincha, Ecuador

**Keywords:** Enfermería, Autonomía Profesional, Rol de Género, Educación en Enfermería, Curriculum.

## Abstract

**Objetivos:**

sintetizar los aspectos claves de la enfermería sobre los que se soportan algunos discursos que determinan su desarrollo.

**Métodos:**

se diseñó una estrategia de búsqueda en dos etapas. Primero, se realizaron búsquedas en base de datos específicas enfocándose en literatura de enfermería. Posteriormente, se identificaron y rastrearon los estudios que abordaran el objetivo de esta revisión. Las técnicas utilizadas fueron el seguimiento de citas, la búsqueda de publicaciones posteriores y el uso de listas de referencias para trabajar en sentido inverso.

**Resultados:**

31 textos fueron analizados y categorizados en cuatro dimensiones: Autonomía profesional; Asuntos de género patriarcal; La escuela de Nightingale; y Reformas curriculares.

**Conclusiones:**

los aspectos sintetizados en esta revisión, a través de los principios que promulgan o de sus discursos ocultos, han procurado el desarrollo profesional y disciplinar de la enfermería o, por el contrario, no permiten que la enfermería sea reconocida socialmente como disciplina profesional.

## INTRODUCCIÓN

La primera lección de Michel Foucault como profesor en el *Collège de France* en 1970 fue “El Orden del Discurso”^(1)^. Durante el desarrollo de esta conferencia magistral, el pensador expresó “… en toda sociedad, la producción del discurso está a la vez controlada, seleccionada y redistribuida por cierto número de procedimientos que tienen por función conjurar sus poderes y peligros, dominar el acontecimiento aleatorio y esquivar su pesada y temible materialidad”^(2)^. En la misma línea de discusión, Foucault^(2)^ menciona que, dentro de todo discurso, se esconden “voluntades de verdad” explicadas por él como las múltiples estructuras o prácticas sociales que crean y sostienen la producción de algún conocimiento, a través de los cuales se transfieren significados y se reflejan sistemas de pensamiento y conocimiento. A partir de los anteriores postulados foucaultianos, la literatura menciona que las formaciones discursivas permiten definir posibilidades conceptuales y determinar con anterioridad, primero, lo que se puede y lo que no se puede decir, y luego, lo que se puede llegar a pensar en un contexto o periodo de tiempo determinado^(3)^.

Adicionalmente, Foucault^(2)^ sostiene que el discurso verdadero atravesó por una “separación histórica”. En el siglo VI, el discurso verdadero -“el que reinaba, el proferido por quien tenía el derecho”- era el discurso que administraba la justicia y que no solo anticipaba los cambios, sino que contribuía con su realización^(2)^. Foucault^(2)^ afirma que, un siglo después, la verdad ya no radicaba en el discurso ni en quien lo provocaba, sino que moraba en lo que el discurso decía -“su sentido, su forma, su objeto”- o a lo que hacía referencia. En este sentido, el discurso podría ya no encontrarse vinculado al ejercicio del poder. Siguiendo a Foucault^(2)^, estas circunstancias históricas -dentro de la producción discursiva- no cesan de desplazarse, provocando descubrimientos que se llegan a constituir y a ser considerados como “nuevas formas de verdad”. Para que se consolide como voluntad de verdad, Foucault^(2)^ menciona que un discurso debe estar apoyado en una “base institucional”. Esta base -en palabras del autor- “está reforzada y acompañada por una serie de prácticas como la pedagogía, el sistema de libros, la edición, las bibliotecas, las sociedades de sabios de antaño, los laboratorios actuales”^(2)^. No obstante, se hace necesario también que esta voluntad responda a una necesidad social para asegurar su apreciación, su distribución y su repartición entre los miembros de una sociedad.

Investigadores de la temática ejemplifican lo anterior desde la academia. Estos autores sostienen que lo que se puede decir sobre el saber enfermero se clasifica según las concepciones subyacentes y los sistemas regulatorios que lo dictaminan y determinan “lo que demuestra una comprensión suficiente dentro del ámbito de la crítica aceptable”^(3)^. Adicionalmente, mencionan que -al igual que otras disciplinas- la enfermería también posee reglas declaradas para conceptualizar sus demandas de conocimiento y, que históricamente, las investigaciones realizadas, desde una perspectiva foucaultiana, han permitido visualizar la configuración interna de lo que se conoce como saber enfermero^(3)^. Para estos autores, es a través de este tipo de investigaciones que ha sido posible el develamiento de los discursos que han sido dominantes en los procesos de formación en enfermería y que han determinado las capacidades y funcionalidades de su ejercicio práctico^(3)^.

Siguiendo a Foucault, el interés por estudiar los discursos -dada su condición de operar bajo la conciencia humana y su capacidad de evidenciar los sistemas de pensamiento y conocimiento que influyen en las prácticas societales^(4)^- se centra en las prácticas que, para este caso, han configurado el desarrollo de la enfermería como disciplina profesional. Bajo esta perspectiva, y de acuerdo con Springer & Clinton^(3)^, las investigaciones de este carácter abordan de manera crítica las complejidades del discurso, su emergencia y resistencia, su contenido, y los contextos que lo determinan, e intentan comprender no solo lo que se sabe de un fenómeno, sino lo que no se expresa de él ya sea por desconocimiento o encubrimiento. En ese sentido, esta revisión busca responder a la interrogante: ¿Cuáles son los aspectos claves de la enfermería sobre los que se sustentan algunos discursos que configuran su desarrollo como disciplina profesional?

## OBJETIVOS

Sintetizar los aspectos clave de la enfermería en los que se basan algunos discursos que determinan su desarrollo.

## MÉTODOS

Se trata de una revisión integradora de la literatura que buscó identificar, analizar y sintetizar resultados de evidencia científica, con el fin de generar conocimiento respecto del tema. Los resultados hacen parte de una tesis doctoral cuyo protocolo fue aprobado por el Comité de Ética de Investigación de la Facultad de Enfermería de la Universidad de Antioquia.

Para llevar a cabo esta revisión, se diseñó una estrategia de búsqueda que siguió las recomendaciones de la adaptación de la declaración *Preferred Reporting Items for Systematic reviews and Meta-Analyses* para el idioma español. Las bases de datos incluidas fueron PubMed, CINALH, JSTOR y Scopus, para el idioma inglés, y la Biblioteca Virtual en Salud y CUIDEN, para el idioma español. El *software* utilizado para la gestión de las referencias fue Mendeley. Los términos de búsqueda incluyeron las palabras claves “*nursing*”, “*education, nursing*”, “*professional autonomy*” y “*gender role*”, haciendo uso del operador booleano “*AND*”. La segunda etapa fue la identificación y rastreo de textos fundamentales que abordaran el objetivo de esta revisión. Las técnicas utilizadas en esta etapa fueron el seguimiento de citas, la búsqueda de publicaciones posteriores y el uso de listas de referencias para trabajar en sentido inverso. Los títulos fueron examinados, inicialmente, para determinar su relevancia. Se realizaron exclusiones en función del objetivo propuesto y de los criterios de selección y exclusión del estudio. Se incluyó literatura en español, portugués e inglés, sin distinción de año de publicación, y se excluyó la bibliografía cuyo texto completo no estaba disponible para su lectura y descarga gratuita, y que se encontraran duplicados. Se identificaron 31 artículos, y su flujo de selección se muestra en la Figura 1.

**Figura 1:** Diagrama de flujo del proceso de selección de estudios según *Preferred Reporting Items for Systematic reviews and Meta-Analyses*, 2022

## RESULTADOS

Se evidencia que la información disponible respecto al tema central de esta revisión corresponde a los últimos 20 años, siendo 2021 el año en el que se ubica el mayor número de publicaciones. La mayor parte de la literatura fue publicada por autores de Brasil y Colombia, destacándose las revistas brasileñas como las que más publicaciones tienen sobre el tema. En cuanto al tipo de estudio, se destacan las reflexiones teóricas y los estudios cualitativos, y también se incluyeron dos estudios transversales. Se incluyeron cuatro capítulos de libro y una tesis que abordan el alcance de esta revisión de manera histórico-crítica. En el Cuadro 1, se especifica lo anterior.

**Cuadro 1:** Descripción de los estudios incluidos en la revisión integradora, 2024

N^o^.	Autores	Título del artículo	Tipo de texto	Categoría	Año
1	Leddy S, Pepper JM^(5)^	Socialización para el ejercicio profesional	Libro	I	1989
2	Romero MN^(6)^	El Modelo Pedagógico en Enfermeria: Una proyección del papel social de la mujer	Reflexión teórica	I	1992
3	Aponte Garzón LH^(7)^	Aspectos pedagógicos en la formación del recurso de enfermería en Colombia	Reflexión teórica	I	1997
4	Cano-Caballero Gálvez MD^(8)^	Enfermería y género Tiempo de reflexión para el cambio	Reflexión teórica	I	2004
5	Varjus SL, Leino-Kilpi H, Suominen T^(9)^	*Professional autonomy of nurses in hospital settings - a review of the literature*	Revisión integrativa	I	2011
6	Watkins C, Hart PL, Mareno N^(10)^	*The effect of preceptor role effectiveness on newly licensed registered nurses perceived psychological empowerment and professional autonomy*	Estudio transversal correlacional	I	2016
7	Velandia Mora AL^(11)^	Historia de la enfermería en Colombia	Libro	I	2016
8	Mena Tudela D, González Chordá VM^(12)^	Imagen social de la enfermería, ¿estamos dónde queremos?	Reflexión teórica	I	2018
9	Oshodi TO, Bruneau B, Crockett R, Kinchington F, Nayar S, West E^(13)^	*Registered nurses’ perceptions and experiences of autonomy: A descriptive phenomenological study*	Análisis fenomenológico	I	2019
10	Franco Coffré JA^(14)^	Percepción social de la profesión de enfermería	Reflexión teórica	I	2019
11	Pursio K, Kankkunen P, Sanner-Stiehr E, Kvist T^(15)^	*Professional autonomy in nursing: An integrative review*	Revisión integrativa	I	2021
12	Webler N, Almeida LCG de, Carneiro JB, Campos LM, Glaeser TA, Couto TM^(16)^	*Professional autonomy in dealing with complications: discourse of obstetric nurses working in planned home births*	Estudio cualitativo	I	2023
13	Matejski MP^(17)^	*Nursing education, professionalism, and autonomy: social constraints and the Goldmark Report*	Reflexión teórica	II	1981
14	Marques Lopes MJ, Cezar Leal SM^(18)^	*A feminização persistente na qualificação profissional*	Reflexión teórica	II	2005
15	Ayala-Valenzuela R^(19)^	Biopoder: el poder y la violencia en la formación de profesionales de enfermería	Reflexión teórica	II	2008
16	Lemos De Souza L, Borges Araújo D, Souza Silva D, Cristina V, Bêrredo M^(20)^	*Representações de gênero na prática de enfermagem na perspectiva de estudantes*	Estudio transversal	II	2014
17	Nogueira I, Spagnol G, Rocha F, Lopes MH, Marques D, Santos D^(21)^	*Gender and Empowerment by Nursing Students: Representations, Discourses and Perspectives*	Análisis de discurso	II	2023
18	Nigthingale F^(22)^	*Notes on nursing: What it is, and what it is not*	Libro	III	1860
19	Cohen B^(23)^	Florence Nigthingale	Reflexión teórica	III	1984
20	Castrillón Agudelo MC^(24)^	La Dimensión Social de la Práctica de la Enfermería: Historia y sociología de la enfermería	Libro	III	2012
21	Riegel F, Crossetti M da GO, Martini JG, Nes AAG^(25)^	*Florence Nightingale’s theory and her contributions to holistic critical thinking in nursing*	Reflexión teórica	III	2021
22	Diogo PMJ, Freitas BHBM de, Costa AIL da, Gaíva MAM^(26)^	*Care in pediatric nursing from the perspective of emotions: from Nightingale to the present*	Reflexión teórica	III	2021
23	Breigeiron MK, Vaccari A, Ribeiro SP^(27)^	*Florence Nightingale: Legacy, present and perspectives in COVID-19 pandemic times*	Reflexión teórica	III	2021
24	McCauley L, Hayes R^(28)^	*From Florence to fossil fuels: Nursing has always been about environmental health*	Reflexión teórica	III	2021
25	Manfredy M^(29)^	Bases teóricas para la estructuración curricular	Libro	IV	1980
26	Organización Panamericana de la Salud^(30)^	Análisis prospectivo de la educación en enfermería	Libro	IV	1989
27	Posada Vera EM^(31)^	Discursos sobre la configuración de la enfermería como disciplina o como profesión: un estudio de caso en una facultad de enfermería	Tesis doctoral	IV	2015
28	Petry S, Padilha MI, Costa R, Mancia JR^(32)^	*Curricular reforms in the transformation of nursing teaching in a federal university*	Estudio de caso único	IV	2020
29	Buchanan C, Howitt ML, Wilson R, Booth RG, Risling T, Bamford M^(33)^	*Predicted Influences of Artificial Intelligence on Nursing Education: Scoping Review*	Revisión de alcance	IV	2021
30	Nogueira IC, Santos D de S, Sanfelice CF de O, Silva EM, Assis AESQ^(34)^	*Gender debate as a challenge in nursing training*	Estudio cualitativo	IV	2021
31	Duque PA, Flórez-Pulido LM, Mejía-Ramírez LF^(35)^	Revisión de literatura integradora del conocimiento disciplinar de la enfermería y el currículo	Reflexión teórica	IV	2024

De los 31 textos seleccionados, emergieron cuatro categorías que engloban los aspectos clave de la enfermería en los que se basan algunos discursos sobre esta profesión. Estos son: Autonomía profesional (12 textos); Asuntos de género patriarcal (5 textos); La escuela de Nightingale (7 textos); y Reformas curriculares (7 textos).

## DISCUSIÓN

### Categoría I - Autonomía profesional

El primer aspecto clave en la enfermería es la búsqueda constante de autonomía profesional, elemento considerado por Leddy & Pepper^(5)^ como fundamental para alcanzar el estatus de profesión. Para Pursio *et al*.^(15)^, la autonomía profesional se relaciona con la participación del personal de enfermería en la toma de decisiones y la capacidad que este tiene de influenciar la práctica laboral. Según esta autora, sobre la autonomía en enfermería, se reconocen dos dimensiones amplias: la autonomía en la práctica clínica y la autonomía en la práctica profesional. Oshodi *et al*.^(13)^ definen la autonomía clínica como “la capacidad para actuar más allá de la práctica estándar y tomar decisiones individuales con respecto a la atención de pacientes”.

Según Varjus *et al*.^(9)^ y Watkins *et al*.^(10)^, la autonomía profesional, además de lo anterior, abarca, respectivamente, la posibilidad que tiene el enfermero de “desarrollar procesos para mejorar la calidad de la atención de enfermería y la seguridad del paciente” y de “su capacidad de influir en las prácticas y condiciones de trabajo”. Pursio *et al*.^(15)^, en su revisión integrativa sobre la autonomía en enfermería, identificaron como factores esenciales para el desarrollo de la autonomía profesional en los enfermeros “el liderazgo compartido, las competencias profesionales, la colaboración inter e intra profesional y un ambiente de trabajo saludable”. Pero, a la vez, menciona que el rol históricamente sostenido de profesión subordinada al gremio médico acrecienta la brecha entre la colaboración interprofesional y fundamenta los roles desiguales en la comunidad laboral.

En esa misma línea de discusión, autoras hispanoamericanas como Aponte^(7)^, Romero^(6)^ y Velandia^(11)^ coinciden en que, detrás del discurso vocacional y religioso de orden moral - que viene dado con el rol de ser mujer -, se esconden relaciones de poder ante instancias consideradas de mayor jerarquía que colocan a la enfermería en una posición permanente de subordinación, situación que interfiere directamente en la realización social, ideológica, educativa y laboral que ha buscado la enfermería por casi dos siglos. Cano^(8)^ identifica como causa de la falta de autonomía en el ejercicio profesional de la enfermería el que los miembros de esta profesión aún no posean una clara definición de los que es el cuidado, y en esa medida, no han entendido ni introyectado las funciones propias e independientes que como disciplina tiene esta profesión. Con esto coinciden Mena & González^(12)^, Franco^(14)^ y Webler *et al*.^(16)^, al afirmar que, para la sociedad - en el ámbito de la asistencia sanitaria -, el médico actualmente es reconocido como la figura masculina que representa fuerza, dominio y conocimiento, y la enfermera sigue siendo estereotipada como la figura femenina, débil, sumisa e invisibilizada que depende del primero para ejercer su profesión. Retomando a Aponte^(7)^ y Romero^(6)^, esta estructura socialmente construida no solo ha regulado el ejercicio de la profesión, sino que ha influido en los procesos de formación, configurando una identidad propia para la enfermería: es femenina, de carácter religioso vocacional, de actuar disciplinado y apegado a la norma, y que responde a un esquema de subordinación hacia el modelo biomédico.

### Categoría II - Asuntos de género patriarcal

Varios autores consideran que los asuntos de género patriarcal son una problemática fundamental y central que continúa ejerciendo influencia sobre la profesión^(16)^. Históricamente, la enfermería se ha asociado al cuidado, y este, a su vez, ha sido concebido desde siempre como una práctica femenina. De Souza *et al*.^(20)^ señalan a Nightingale como responsable de la instauración de la enfermería moderna bajo principios morales y religiosos que se han perpetuado hasta la presente y que han promovido la división sexual del trabajo desde mediados del siglo XIX. Para estos autores, esta división sexual va de la mano de la división social del trabajo entre medicina y enfermería, puesto que esta diferenciación se asemeja a la existente entre la ciencia y el arte, el estudio y la vocación, y el trabajo intelectual y el trabajo manual, diferencias que no se relacionan únicamente a los asuntos de género patriarcal, sino que también hacen alusión a asuntos de raza, clase, religión, edad y demás componentes del tejido social^(20)^.

Foucault^(36)^ - filósofo del discurso y del poder que se ejerce detrás de el - sostiene que el poder se practica a través de las relaciones y que este no solo tiene carácter dominante, sino que se exhibe como un poder productivo dirigido al cuerpo para entrenarlo y moldearlo a su manera. Siendo así, los asuntos de carácter religioso, de género, clasismo y cultura patriarcal, que históricamente han permeado a la profesión de enfermería, tienen alta responsabilidad en la configuración de este saber y de su ejercicio. Así lo demuestra De Souza^(20)^, en su estudio observacional sobre discursos de género en estudiantes de enfermería, en el cual identificó el uso de un lenguaje cotidiano en sus relaciones sociales que propaga estereotipos de género y que los representa como auxiliares médicos autoritarios, bondadosos, sumisos, angelicales, devotos, y otros de mayor tono, como prostituta u homosexual. Para Marques & Cezar^(18)^, la dicotomía perpetua entre la práctica de la medicina (tratamiento) y la práctica de la enfermería (cuidado) demuestra claramente el poder de los hombres sobre las mujeres, materializado en el poco reconocimiento que tiene la enfermería en el ámbito social, en la aplicación de jornadas laborales más largas y en la imposición de salarios más bajos. En la misma línea, Nogueira *et al*.^(21)^ sostienen que estas prácticas, además de fortalecer los estereotipos de género y la marginación de los profesionales de enfermería, contribuyen a la extensión del modelo biomédico del sistema neoliberal hegemónico, que está más preocupado por tratar las enfermedades que por reorientar sus esfuerzos hacia el desarrollo de una sociedad más saludable.

Como se ha visto, el desarrollo de la enfermería como profesión y disciplina ha estado sujeto a las relaciones de poder que han transversalizado este proceso por más de 200 años. Según Ayala^(19)^, en la naturaleza, el poder se manifiesta de diversas formas y se propaga a través de las relaciones humanas clásicas de: hombre-mujer, padre-hijo, médico-enfermo, profesor-estudiante, etc. Retomando a Foucault^(37)^, este autor define al poder como “las múltiples formas de dominación que pueden ejercerse en el interior de la sociedad”, e identifica los espacios académicos o educacionales, los hospitales, las iglesias y las cárceles^(36)^ como nichos insuperables para el ejercicio y reproducción de las relaciones de poder. A pesar de constituirse como esferas cuyas dinámicas son totalmente diferenciadas, las relaciones de poder que se dan en los espacios anteriormente nombrados se consolidan, legitiman y perpetuán desde una lógica similar: las prácticas discursivas^(19)^. Sobre lo anterior, Ayala^(19)^ menciona que la diferenciación entre las dinámicas de los espacios de poder está determinada por dos factores: primero por las relaciones interpersonales entre los miembros que hacen parte de ese colectivo (escuela, hospital, cárcel, etc.); segundo por el carácter de institución cerrada bajo el cual se crean dichos espacios. En la misma línea, Deleuze^(38)^ - otro discípulo de Foucault - sostiene que el ser humano transita durante toda su vida a través de relaciones de poder: primero dentro de la familia, luego en el interior de la escuela, si es hombre dentro del cuartel, de vez en cuando el hospital, y en algunos casos la prisión^(38)^.

Según Foucault^(36)^, en las relaciones de poder, cobra énfasis el dominio y manipulación intencional del cuerpo del hombre “al que se le da forma, se le educa, el que obedece, responde, se vuelve hábil o cuyas fuerzas se multiplican”. Ayala^(19)^ - en sus reflexiones sobre el biopoder - sostiene que, desde la época clásica, el cuerpo fue objeto de estudio con el fin de “conocerlo, comprenderlo y hacerlo inteligible”. Ya desde una mirada biopolítica, Foucault^(36)^ estudia el cuerpo desde una lógica de sometimiento, ejercido a través de normas militares, escolares u hospitalarias, establecidas para controlar o intervenir las prácticas operacionales de este. Para Ayala^(19)^, la educación ejerce un papel fundamental en el modelado de los cuerpos, aplicando sus normas disciplinarias en un contexto permanente de coerción. En palabras de Foucault^(36)^, “el control disciplinario no consiste simplemente en enseñar o en imponer una serie de gestos definidos; impone la mejor relación entre un gesto y la actitud global del cuerpo, que es su condición de eficacia y de rapidez”. Para ejemplificar la presencia histórica de este tipo de prácticas en los procesos de enseñanza en enfermería, Matejski^(17)^ - en su investigación sobre educación, profesionalismo, y autonomía en enfermería - resalta un fragmento del relato sobre los primeros 40 años de funcionamiento de la Escuela de Enfermería del Hospital Johns Hopkins: “La enfermera es un soldado. La obediencia absoluta e incondicional es la idea fundamental del sistema militar... el rigor y la exactitud producen mejores enfermeras”.

### Categoría III - La escuela de Nightingale

Con la creación del primer programa de formación para enfermeras, dirigido por Florence Nightingale, se instauró un modelo de formación que - a decir de varios autores - aún se evidencia en algunos currículos de la región y que interviene, de manera permanente, en la consolidación de la enfermería como disciplina profesional. Cohen^(23)^ al respecto menciona: “El objetivo de esta escuela era formar enfermeras capaces de adiestrar a otras. Las enfermeras que se formarían en esta escuela no se iban a dedicar al cuidado de enfermos, sino que irían a dirigir los hospitales e instituciones públicas, a fin de darle un estatus más alto a la atención de enfermería”.

Según este autor, la escuela nightingaleana, creada en 1859, se regía por tres principios: primero, las aspirantes a enfermeras recibían un año de formación y entrenamiento técnico en hospitales; segundo, dichas estudiantes debían vivir en una residencia organizada para supervisar de cerca su moral y disciplina; y tercero, esta residencia debía estar cerca del hospital, para que las estudiantes estuvieran disponibles para prestar servicios en cualquier momento, y así ellas pudieran aprender haciendo^(23)^. Para Castrillón^(24)^, a pesar de parecer principios radicales propios de esa época, en muchas escuelas, estos principios se mantuvieron y perpetuaron por más de un siglo, o al menos hasta la instauración de un sistema de formación profesional para la enfermería. Sin embargo, Riegel *et al*.^(25)^ sostienen que el legado de Nightingale es decisivo para llenar los vacíos de conocimiento relacionados con la dimensión holística del cuidado de enfermería, lo que redunda en una toma de decisiones clínicas efectivas por parte de los estudiantes. Breigeiron *et al*.^(27)^ y Diogo *et al*.^(26)^ coinciden con esto y concluyen que este modelo fue base para la consolidación de la profesión de enfermería.

Otro de los legados discursivos que configuraron la práctica enfermera fueron las famosas notas de enfermería de Florence Nightingale. En dichas notas, la autora define a la enfermería como “el arte de mantener el estado de la salud, previniendo la enfermedad o recuperándose de ella”^(22)^. Sobre esto, hay una teoría establecida, la teoría ambiental o del entorno, en la que, según McCauley & Hayes^(28)^, se describen diez factores que influyen en la salud y que deben ser tomados en consideración en los cuidados de enfermería. Adicionalmente, estas notas también reconocen la necesidad de crear centros de formación para formar personal especializado, tanto para la atención de pacientes hospitalizados como para aquellos que requieren otro tipo de cuidados especiales, como los ancianos y las personas con discapacidad^(22)^. Para Castrillón^(24)^, este modelo de pensamiento y de formación particular ha dejado un legado perpetuo en la profesión de enfermería, traducido en un mayor número de enfermeras y enfermeros que ejercen en el ámbito asistencial hospitalario - propio de un modelo sanitario con mayor énfasis en lo curativo - y que representa el ideal de gran parte de los profesionales en formación.

### Categoría IV - Reformas curriculares

Según Castrillón^(24)^, no existe otra escuela universitaria con mayor número de transformaciones curriculares - producto del influjo discursivo de políticas públicas y educativas - como la de enfermería. Para esta autora, la enfermería se constituye en la práctica más susceptible al recibir y entregar funciones y actividades en respuesta a las transformaciones relacionadas con la división del trabajo en el sector sanitario, y el permanente ingreso de personal técnico o no profesional^(24)^. Castrillón^(24)^ tiene la concepción de que los modelos de formación en enfermería se preocupan más por la producción de servidores públicos que por la preparación de profesionales.

Manfredy^(29)^ definió cuatro etapas que caracterizaron la educación en enfermería en el Latinoamérica entre 1940 y 1980. Esta autora señala que, en la década de los cuarenta, los planes de estudios desarrollados para esta formación estaban estructurados por cátedras aisladas, no evidenciaban una relación entre el sector sanitario y educación superior, y se aplicaban a través del método de la conferencia magistral, cuyo fin era replicar la imagen del profesor en el estudiante en formación^(29)^.

Ya en la década de los 50, en esta región: los modelos de formación se caracterizaron por la división de la estructura curricular entre asignaturas básicas y clínicas; los niveles de enseñanza se organizaron anualmente; y la metodología -muy similar a la anterior- se combinó con la enseñanza demostrativa mediante el uso de simuladores o en laboratorios acondicionados para tal fin^(29)^. Desde inicios de la década de los 60, a raíz de un nuevo cambio en la estructura curricular, la distribución de asignaturas respondió al estudio del cuerpo fraccional, incorporándose la enseñanza de las ciencias sociales y humanísticas. Adicionalmente, surge el interés por equipar las bibliotecas de las escuelas con material y guías de práctica clínica y comunitaria contextualizadas para la región^(29)^. Finalmente, entre 1970 y 1980, las modificaciones curriculares respondieron a dos fenómenos: primero, existe la preocupación de construir un perfil profesional adecuado a las necesidades de cada país de la región, y no sólo en materia de salud, sino también de salud familiar y colectiva, es decir, de atención primaria de salud; y segundo, incluye el desarrollo de perfiles, a través de los cuales se puede lograr la integración docencia-asistencial para el desarrollo de prácticas clínicas y comunitarias^(29)^.

Castrillón^(24)^ avanza un poco más con el análisis de las transformaciones curriculares en enfermería hasta finales del siglo XX. Para esta autora, a partir de los 80, y gracias al movimiento generado por la declaración de Alma Ata de 1978, se pusieron en marcha algunas estrategias para fortalecer los procesos de formación profesional en enfermería^(24)^. Destacan, entre otras, reorientar la enseñanza de las ciencias sociales en función de la realidad sociosanitaria del país; innovar las prácticas clínicas; fortalecer las prácticas de promoción de la salud comunitaria; y potencializar el desarrollo de habilidades relacionadas con la valoración física. En relación a la planificación y ejecución de la atención que garantice la atención integral de la salud, en definitiva, según palabras de Castrillón^(24)^, “se buscaba una formación integral que tuviera como marco de referencia para la estructura curricular, la situación del país”. Para finales de los 90 - en el marco de la creación de políticas propias para la educación superior -, la misma autora menciona que las universidades iniciaron un proceso de transformación, en la búsqueda de, ya no solo aportar a la sociedad a través de la docencia, sino a través de la generación de nuevos conocimientos como producto de la investigación científica^(24)^.

Particularmente en la enfermería, estas transformaciones recientes dieron lugar a la generación de líneas de investigación que posteriormente sustentaron: la creación de grupos para desarrollarlas y presentarlas; la necesidad de crear cursos de posgrado específicos para el desarrollo de la enfermería; la participación en proyectos multidisciplinarios; y el establecimiento de convenios marco no sólo para ampliar escenarios de práctica clínica, sino también para el intercambio de experiencias académicas tanto de docentes como de estudiantes^(24)^. En palabras de Castrillón, “todo esto fue reconfigurando los procesos docentes-asistenciales”.

La última década del siglo XX se caracterizó por la puesta en marcha de planes de intervención no solo a los planes de estudios, sino a la infraestructura de las escuelas, como producto del análisis retrospectivo realizado - bajo la asesoría de la Organización Panamericana de la Salud (OPS) - a varias escuelas de enfermería de diez países latinoamericanos^(30)^. Las intervenciones propuestas incluían el desarrollo de proyectos asistenciales-docentes e investigaciones que ayudaran a solventar las problemáticas locales de la salud y de la profesión de enfermería; la necesidad de constituir redes académicas internacionales en donde los actores principales de la profesión se vincularan a través del debate académico sobre la construcción del saber enfermero; y los aspectos que lo circundan^(30)^. Adicionalmente, se hacía un llamado a incrementar el número de publicaciones científicas, a la participación en eventos académicos, y al desarrollo de programas de educación continua para graduados. Según Posada^(31)^, todos los elementos anteriormente nombrados son determinantes para la consolidación de la enfermería como disciplina profesional. En ese sentido, siguiendo a Castrillón^(24)^, en América Latina, este proceso se empezó a evidenciar para enfermería a partir de los 90.

Castrillón^(24)^ hace una reflexión final, aseverando que la OPS ha jugado un papel central en la propuesta y promulgación de políticas o guías sobre las cuales se han fundamentado la mayoría de dichas transformaciones curriculares. Esta autora también sostiene que estas variaciones en los currículos no se han dado de forma simultánea, o desde las mismas perspectivas a nivel regional y mundial, y que esto es producto de los múltiples debates, interpretaciones o incluso resistencias que el tema genera^(24)^. Sobre esto, Petry *et al*.^(32)^ aseguran que la estructura curricular y las reformas que se efectúan de manera constante para la formación en enfermería se configuran a la par del contexto histórico, político, epidemiológico y social que necesita la profesión para satisfacer las demandas de la sociedad y el mercado laboral.

Un ejemplo de lo anterior es el contexto pospandémico en que se encuentra la sociedad actual. Para Buchanan *et al*.^(33)^, urge una reforma curricular en los programas de formación de enfermería que incluya en sus perfiles de egresos las habilidades y destrezas para ejercer una práctica de cuidado segura y eficiente en el marco de la era digital y el uso de la inteligencia artificial en los sistemas de salud. Adicionalmente, Duque *et al*.^(35)^ sostienen que es primordial que los currículos se permeen del metaparadigma disciplinar para guiar los procesos de enseñanza y aprendizaje hacia la construcción de un perfil enfermero que obtenga mayor visibilidad, y que respalde su práctica en el método científico y en los patrones de conocimiento propios de la enfermería. Finalmente, y en correspondencia con las categorías antes enunciadas, Nogueira *et al*.^(34)^ aseguran que la transformación de los procesos de formación en enfermería es urgente y que, en la actualidad, también se debe considerar, entre los ejes transversales del currículo, el análisis de los asuntos de género y su influencia directa en el ejercicio profesional.

### Limitaciones del estudio

La principal limitación de esta revisión se instala en la cobertura geográfica de los estudios seleccionados. Haría falta explorar la temática del desarrollo de la enfermería y los aspectos que lo configuran desde el contexto anglosajón y asiático en donde actualmente la producción de conocimiento en enfermería es considerable.

### Contribuciones al área de enfermería, salud o políticas públicas

Según Foucault, los discursos enmascaran una lógica de poder que, así como permite, impide. En este sentido, sintetizar los aspectos claves de la enfermería sobre los cuales se soportan algunos discursos en esta profesión se convierte en un marco de referencia para identificar los elementos que se requiere intervenir para procurar un mayor desarrollo profesional y disciplinar de la enfermería, e identificar aspectos de la formación que podrían modificarse para el mismo fin.

## CONCLUSIONES

La revisión bibliográfica revela algunos aspectos en los que -desde los orígenes de la enfermería moderna- se han fundamentado algunos de los discursos que se han implementado y circulado a lo largo de la historia de la enfermería, siendo determinantes en los procesos de formación y en la difusión del conocimiento en enfermería. Entre tantos, la literatura destaca la búsqueda de autonomía profesional, las cuestiones patriarcales de género, la escuela de Florence Nightingale y las reformas curriculares que, por los principios que promulgan o por sus intereses ocultos, han buscado el desarrollo profesional y disciplinar de la enfermería o, por el contrario, han impedido que la enfermería se posicione y sea reconocida socialmente como disciplina profesional.

## Data Availability

Los datos de la investigación están disponibles en un repositorio: https://preprints.scielo.org/index.php/scielo/preprint/view/11144/version/11750.
